# Migrant health in French Guiana: Are undocumented immigrants more vulnerable?

**DOI:** 10.1186/1471-2458-12-53

**Published:** 2012-01-19

**Authors:** Anne Jolivet, Emmanuelle Cadot, Sophie Florence, Sophie Lesieur, Jacques Lebas, Pierre Chauvin

**Affiliations:** 1 INSERM, U707, Research Team on the Social Determinants of Health and Health Care, Faculté de médecine Pierre et Marie Curie, site St-Antoine, 27 Rue de Chaligny, 75571 Paris Cedex 12, France; 2 Université Pierre et Marie Curie - Paris 06, UMR-S 707 Paris, France; 3 AP-HP, Hôpital Saint-Antoine, Paris, France

**Keywords:** Immigrant, Undocumented migrant, French Guiana, Health status, Cross-sectional survey

## Abstract

**Background:**

Few data exist on the health status of the immigrant population in French Guiana. The main objective of this article was to identify differences in its health status in relation to that of the native-born population.

**Methods:**

A representative, population-based, cross-sectional survey was conducted in 2009 among 1027 adults living in Cayenne and St-Laurent du Maroni. Health status was assessed in terms of self-perceived health, chronic diseases and functional limitations. The migration variables were immigration status, the duration of residence in French Guiana and the country of birth. Logistic regression models were conducted.

**Results:**

Immigrants account for 40.5% and 57.8% of the adult population of Cayenne and St-Laurent du Maroni, respectively. Most of them (60.7% and 77.5%, respectively) had been living in French Guiana for more than 10 years. A large proportion were still undocumented or had a precarious legal status. The undocumented immigrants reported the worst health status (OR = 3.18 [1.21-7.84] for self-perceived health, OR = 2.79 [1.22-6.34] for a chronic disease, and OR = 2.17 [1.00-4.70] for a functional limitation). These differences are partially explained by socioeconomic status and psychosocial factors. The country of birth and the duration of residence also had an impact on health indicators.

**Conclusion:**

Data on immigrant health are scarce in France, and more generally, immigrant health problems have been largely ignored in public health policies. Immigrant health status is of crucial interest to health policy planners, and it is especially relevant in French Guiana, considering the size of the foreign-born population in that region.

## Background

A growing body of studies suggests that there are health disparities between immigrants and local populations [1-4]. Despite growing knowledge, the relationship between migration and health remains complex and dynamic, for many migration-related determinants can have an impact on health [3]. French studies, too, have reported that disparities in health outcomes exist between immigrants and native-born individuals [5-8], although such studies are rare in France, where categorizing people as immigrants is viewed as a sensitive issue and is governed by strict legal rules.

French Guiana is located in a humid equatorial zone of South America, between Brazil to the southeast and Surinam to the northwest. A former French colony, French Guiana became, in 1946, a French overseas territory, with the same legislation as in mainland France. French Guiana has a multiethnic population, the result of successive migration waves. Up until the early 1960s, the history of French Guiana was characterized by problematic and insufficient human settlement (in 1954, the population was still only 27,000, over an area of 83,350 km^2^, essentially Creoles, Amerindians and Bushinenge). It subsequently attracted a great deal of foreign labour enticed by a job market that had become attractive with the creation of the Guiana Space Centre and the launch of large infrastructure projects. In the 1970s and 1980s, it took in a large number of migrants fleeing from the political instability and economic hardships in their countries: political turmoil in Haiti, a civil war in Surinam (1986-1992), and social and economic problems in Guyana. French Guiana has been going through a major economic crisis since the 1990s, with a high unemployment rate (20.6% of the active population in 2006), a huge trade deficit and heavy economic dependence on public transfers [9]. Despite this economic crisis and increasingly restrictive immigration policies, there is still significant migratory pressure. In 2009, this department had 229,000 inhabitants, 29.5% of whom were immigrants [9]. There are few data on the health of this immigrant population.

The objective of this article is to analyze health disparities between immigrants and native-born people in light of several migratory characteristics (the immigrants' legal status, their duration of residence in French Guiana, and their country of origin).

## Methods

### Study design

A representative, population-based, cross-sectional survey was conducted in French Guiana's two largest cities: Cayenne and Saint-Laurent du Maroni, which had 58,004 and 33,707 inhabitants, respectively, as at January 1, 2006 [9]. The target population consisted of the resident adult population (≥ 18 years), "resident" meaning having lived or intending to live in either of these two cities for at least 6 months.

**Figure 1:**
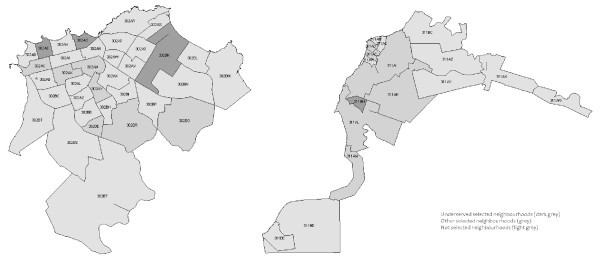
**Map of the randomly selected neighbourhoods in Cayenne and Saint-Laurent du Maroni**.

A four-stage random sample was constituted. The objective was to conduct 600 interviews in Cayenne and 400 in Saint-Laurent du Maroni (in order to respect the population ratio between the two cities) and to interview 60 people per neighbourhood. These neighbourhoods constitute an intermediate aggregated geographical level between residential IRIS [10] (IRIS, a French acronym for "blocks for incorporating statistical information", are aggregated census blocks) and census blocks. First, 10 neighbourhoods were selected from the 34 neighbourhoods in Cayenne (which has 25 IRIS) and 7 were selected from the 17 in Saint-Laurent du Maroni (10 IRIS) in proportion to the number of households (according to the 2009 census), and they were stratified according to whether or not they are designated as "underserved neighbourhoods" by French urban public policies (Figure 1). Second, in each neighbourhood, census blocks were selected proportionally to the number of households. In all, 40 census blocks were randomly selected from the 474 eligible census blocks in Cayenne, and 25 were randomly selected from the 160 in Saint-Laurent du Maroni. Subsequently, households were randomly selected using a sampling interval calculated for each block in proportion to the number of households in that block (the sampling interval varied between 1 and ¼). Lastly, one adult within each household was randomly selected by the interviewer. The questionnaire was administered face-to-face at the individuals' residences by local, multilingual interviewers from February to April 2009. This survey did not fall into the category of biomedical research (as defined by French law) and did not collect any personal identification data. Therefore it did not need ethical approval in France. On the other hand, it has been approved by the Department of research of the Agence française de développement (AFD).

### Data collection

#### Health status

We used the three health-related questions from the Mini European Health Module (MEHM) that concern self-assessed health, chronic diseases and functional limitations [11,12]:

• *Self-assessed health *was based on the question, "How would you describe your general health?", to which the possible answers were "very good", "good", "fair", "poor" and "very poor". This indicator was dichotomised between the individuals who assessed their overall health as very poor, poor or fair and those who assessed it as good or very good.

• *Chronic disease status *was assessed by the question, "Do you have any longstanding illness or longstanding health problem?", "longstanding" referring to illnesses or health problems that had lasted or were expected to last for 6 months or longer.

• *Functional limitations *were assessed by the question, "For at least the past six months, have you been limited because of a health problem in activities people usually do?"

#### Migration variables

Three variables were examined:

1) *Migration status *was defined on the basis of four variables: the country of birth, nationality at birth, nationality on the day of the interview and, for those of foreign nationality, their legal status on the day of the interview. Six migration statuses were thus defined:

- Native-born French. Applies to people of French nationality born in French Guiana. They were chosen as the reference category.

- Born French outside French Guiana (in mainland France, another French overseas territory or abroad). Such individuals were differentiated from the previous group in that they constitute a special subgroup (migration is often temporary, and they often enjoy a privileged socioeconomic status).

- Naturalized immigrant. Applies to people who had acquired French citizenship.

- Long-term documented immigrant. Applies to migrants of foreign nationality who had a 10-year French territory residence card. The few citizens of the European Union were included in this subgroup.

- Temporary documented immigrant. Applies to migrants of foreign nationality with a 1-year temporary stay document, authorization for a temporary stay (usually 6 months) or, more rarely, a refugee claim in progress.

- Undocumented immigrants. Applies to migrants of foreign nationality who had no valid stay document on the day of the interview.

Immigrants (born non-French abroad) are therefore represented by the last four categories.

2) *Duration of residence*. In addition, immigrants were classified into two groups according to their duration of residence in French Guiana: ≤ 5 years (recent immigrant) or > 5 years (established immigrant).

3) *Country of birth*. In light of the sample size limitations, the analyses concerned only the two main groups of immigrants: those born in Haiti and those born in Surinam.

#### Covariables

The demographic variables included *gender *and *age*. Median age and the interquartiles were calculated for the description of the population, and four categories ([18-30 years], [30-40 years], [40-50 years] and > 50 years) were used in logistic regression models.

Socioeconomic status was characterized by three variables: *education level*, of which there were three categories (none or primary, secondary and tertiary); *occupational status*, which was categorized as civil servant, upper white-collar, lower white-collar, blue-collar (including farmer), unemployed, housewife, student, retired, and inactive; and *perceived financial situation*. The latter was assessed by a question put to the head of the household ("Presently, for this household, would you say that financially..."), for which there were five possible answers ("We don't have enough to live on; we can't get by.", "We have just enough to live on, but we go without a lot of things", "We have enough to live on as long as we're careful.", "We aren't lacking for anything important.", and "We don't go without anything at all; we're very well off."). This variable was divided into three categories: good (the last two answers), fair and poor (the first two answers). Lastly, two binary psychosocial variables were taken into account. One was *fluency in French *(fluent in French, with no difficulty or with some difficulty, versus not fluent in French at all, with a great deal of difficulty). This variable provided an indication of acculturation to French society. The other one was *feeling of loneliness*, which was assessed by the question, "In general, would you say that you...?" ("have a very good circle of people around you" or "have a fairly good circle of people around you" versus "feel fairly alone" or "very alone").

### Statistical analyses

All of the following analyses were weighted in order to account for the sample design and the poststratification adjustment for age, gender and citizenship status (French or foreigner) according to the general population census performed in 2006 by the Institut National de la Statistique et des Études Économiques (the French Bureau of Statistics).

First, we described and compared the demographic characteristics, socioeconomic conditions and health status of the migration status subgroups using a chi-square test. The comparisons of the median durations of residence and age used the nonparametric test of Kruskal-Wallis. Second, we performed logistic regression models, which were systematically adjusted for age and gender, to estimate the associations between the above-mentioned covariables and each of the three health status variables. Third, we compared the odds ratio (OR) estimating the strength of the association between each of the three migration variables and each of the three health status variables separately when successively adding the covariables to the respective models. Fourth, we constructed a new variable - migration status and origin - that combined the undocumented immigrants' migration status and country of birth, and, in the same manner as in step 3, we determined whether the covariables contributed to the associations observed between this migration status-and-origin variable and each of the three health status variables. All the analyses were performed with Stata^® ^software, version 10.0.

## Results

In all, 1027 people were interviewed (607 in Cayenne and 420 in Saint-Laurent du Maroni). The participation rate was 81.2%. Of the study population, 52.9% were women, and the median age was 36 years (Table 1). The distribution of this population by migration status was as follows: 37.8% were born in French Guiana and were of French nationality; 16.1% were born French outside French Guiana (70.9% of them were born in mainland France); 6.9% were naturalized immigrants (more than half were from the Caribbean, and the median duration of residence was 25 years); 14.2% were long-term documented immigrants and 11.0% were temporary documented immigrants (the median duration of residence was 21 years and 9 years, respectively. These two subgroups consisted mostly of people from Haiti, Surinam and Brazil); and lastly, 14.0% were undocumented immigrants (half of this subgroup were from Surinam, and the median duration of residence was 9 years). It is also worth noting that the age and gender distributions of the native-born French and the undocumented immigrants were quite similar to each other and to the overall distribution, as compared to other migration status groups. A comparison of the socioeconomic conditions according to these six migration profiles showed strong disparities. For the people who perceived their financial situation as having enough to live on, the civil servants and the people who had a higher education, we observed a socioeconomic gradient based on the following six migration profiles: those born French outside French Guiana were always in a more favourable situation, followed by the native-born, naturalized immigrants, long-term documented immigrants and temporary documented immigrants, in that order, with, at the very bottom of this gradient, undocumented immigrants, who were in the most unfavourable socioeconomic circumstances. People who were fluent in French followed an exactly identical gradient.

**Table 1:** Description of the population by migration status

		Native -born French	Born French outside French Guiana	Naturalized immigrant	Long-term documented immigrant	Temporary documented immigrant	Undocumented immigrant	Total	p
		**%**	**%**	**%**	**%**	**%**	**%**	****%****	

**Gender**	Female	52.5	46.8	60.1	49.7	63.3	52.2	**52.9**	0.38

	Male	47.5	53.2	39.9	50.3	36.7	47.8	**47.1**	

**Age**: Median [Interquartiles]		35 [25-49]	38 [31-50]	47 [32-56]	47 [38-53]	32 [23-41]	30 [24-38]	**36 [26-48]**	**<10**^**-3**^

**Country of birth**	French Guiana	100	-	-	-	-	1.7	**38.1**	**<10**^**-3**^

	Mainland France	-	70.9	-	-	-	-	**11.4**	

	Other French overseas territory	-	19.4	-	-	-	-	**3.1**	

	Haiti	-	-	29.7	40.8	34.8	33.0	**16.3**	

	Surinam	-	2.1	6.9	19.6	27.3	50.1	**13.6**	

	Brazil	-	1.6	5.1	19.4	17.4	3.0	**5.7**	

	Other South American country	-	-	15.3	9.1	7.4	6.4	**4.1**	

	Other Caribbean country	-	2.6	23.0	4.8	11.5	5.4	**4.7**	

	Asia	-	-	9.2	4.3	1.7	0.4	**1.5**	

	Other	-	3.5	10.8	2.0	0	0	**1.6**	

**Duration of residence**: Median [Interquartiles]		-	5 [1-13]	25 [18-31]	21 [17-27]	9 [5-16]	9 [5-16]	**16 [7-23]**	**<10**^**-3**^

**City**	Cayenne	77.4	72.9	80.2	65.7	65.7	41.7	**69.0**	**0.04**

	Saint-Laurent du Maroni	22.6	27.1	19.8	34.3	34.3	58.3	**31.0**	

**Educational level**	None or primary	12.6	6.2	25.9	41.1	20.0	35.6	**20.6**	**<10**^**-3**^

	Secondary	66.0	41.5	52.5	53.5	76.1	61.2	**59.8**	

	Tertiary	21.4	52.3	21.7	5.4	4.0	3.2	**19.7**	

**Occupational status**	Civil servant	13.0	38.9	5.0	2.0	-	-	**11.8 **	**<10**^**-3**^

	Upper white-collar	5.7	14.6	-	0.4	-	-	**4.6**	

	Lower white-collar	21.4	12.0	27.0	27.3	19.7	8.3	**19.1**	

	Blue-collar	14.2	6.6	10.5	20.8	16.4	40.8	**17.5**	

	Unemployed	10.7	8.8	8.3	18.1	20.2	0.8	**10.9**	

	Homemaker	8.0	5.6	24.9	17.3	21.8	39.5	**15.9**	

	Student	8.1	1.0	1.7	-	15.5	3.2	**5.4**	

	Retired	14.0	11.4	16.6	9.2	2.4	0.4	**10.0**	

	Inactive	5.0	1.1	7.6	4.8	4.0	7.0	**4.7**	

**Perceived financial situation**	Good	30.8	59.4	32.3	11.8	9.8	4.6	**20.6**	**<10**^**-3**^

	Fair	35.5	26.5	34.9	38.5	32.3	29.4	**34.5**	

	Poor	33.7	14.1	32.9	49.8	57.9	66.1	**44.8**	

**Fluency in French**	Good	97.2	100	82.3	71.7	68.9	46.1	**82.8**	**<10**^**-3**^

	Fair	2.8	-	17.7	28.3	31.1	54.0	**17.2**	

**Feeling of loneliness**	No	87.1	81.5	73.7	83.2	78.9	73.6	**82.0**	0.1

	Yes	12.9	18.5	26.3	16.8	21.1	26.4	**18.0**	

**Health status**	Poor self-assessed health	35.3	19.4	52.3	50.9	48.4	47.0	**39.2**	**0.008**

	Chronic disease	22.4	15.4	44.7	35.0	22.7	33.4	**26.2 **	**0.004**

	Functional limitation	17.9	10.0	32.2	27.0	13.0	19.8	**18.6 **	**0.02**

**Total**		**37.8**	**16.1**	**6.9**	**14.2**	**11.0**	**14.0**	**100**	

All comparisons used Chi2 Test, except comparisons of ages and durations of residence, which used the Kruskal-Wallis test

Since advanced age and female sex were associated with poorer health status indicators, the rest of the analyses were systematically adjusted for these two demographic variables. Table 2 shows that, after such an adjustment, the characteristics significantly associated with poorer health were (regardless of the health variable) being an unemployed, retired or some other inactive individual, being in a poorly perceived financial situation, feeling socially isolated, and having poor fluency in French. Being a homemaker and having a low education level were associated with poorer perceived health and a reported functional limitation, but not of a chronic disease. The blue-collars and lower white-collars were more likely to report poor perceived health.

**Table 2:** Logistic regression models analyzing the health variables according to the demographic, socioeconomic and psychosocial variables (OR and 95% CI)

		Poor self-assessed health		Chronic disease		Functional limitation	
		**%**	**OR (95% CI)**	**%**	**OR (95% CI)**	**%**	**OR (95% CI)**

**Gender**	Male	34.1	1	21.2	1	16.1	1
	
	Female	44.0	**1.52 (1.10- 2.11)**	30.2	**1.61 (1.06- 2.46)**	20.6	1.35 (0.87- 2.10)

**Age**	[18-30 years]	27.5	1	15.2	1	6.3	1
	
	[30-40 years]	32.7	1.28 (0.77- 2.15)	19.1	1.32 (0.78- 2.25)	17.2	**3.10 (1.54- 6.27)**
	
	[40-50 years]	40.9	1.83 (0.99- 3.38)	25.1	**1.87 (1.02- 3.44)**	18.0	**3.29 (1.06- 10.19)**
	
	> 50 years	64.2	**4.74 (2.59- 8.68)**	51.4	**5.92 (3.44- 10.17)**	39.6	**9.79 (3.84- 24.99)**

		**%**	aOR* (95% CI)	**%**	aOR* (95% CI)	**%**	aOR* (95% CI)

**Education level**	Tertiary	12.7	1	18.3	1	7.6	1
	
	Secondary	38.6	**4.32 (2.52- 7.38)**	21.3	1.10 (0.62- 1.93)	14.9	**2.16 (1.05- 4.42)**
	
	None or primary	68.8	**10.72 (4.69- 24.49)**	47.8	2.42 (0.89- 6.53)	39.4	**4.69 (2.58- 8.53)**

**Occupational status**	Civil servant	14.7	1	17.8	1	8.8	1
	
	Upper white-collar	10.7	0.78 (0.23- 2.64)	5.0	0.28 (0.06- 1.26)	15.6	2.38 (0.64- 8.87)
	
	Lower white-collar	36.6	**4.25 (1.86- 9.73)**	18.2	1.33 (0.48- 3.66)	9.5	1.31 (0.49- 3.51)
	
	Blue-collar	38.5	**5.74 (3.20- 10.28)**	24.8	2.17 (0.92- 5.11)	10.3	1.45 (0.54- 3.91)
	
	Unemployed	39.2	**4.74 (2.29- 9.85)**	26.3	**2.07 (1.03- 4.15)**	14.9	**2.76 (1.27- 5.99)**
	
	Homemaker	48.9	**5.57 (2.74- 10.33)**	28.9	1.79 (0.72- 4.45)	28.6	**5.42 (2.35- 12.51)**
	
	Student	21.5	3.37 (0.70- 16.28)	13.2	1.59 (0.29- 8.59)	1.2	0.46 (0.07- 3.07)
	
	Retired	77.5	**11.59 (2.62- 51.29)**	59.8	**3.07 (1.11- 8.46)**	50.7	**6.30 (2.14- 18.59)**
	
	Inactive	54.0	**10.06 (2.46- 43.9)**	39.1	**4.15 (1.03- 16.70)**	38.3	**10.73 (3.81- 30.22)**

**Perceived financial situation**	Good	26.0	1	16.1	1	10.2	1
	
	Fair	40.3	1.81 (0.79- 4.15)	26.7	1.74 (0.96- 3.16)	17.3	1.58 (0.72- 3.50)
	
	Poor	47.9	**2.47 (1.12- 5.43)**	32.6	**2.22 (1.16- 4.26)**	25.5	**2.70 (1.29- 5.67)**

**Fluency in French**	Good	35.6	1	23.6	1	15.2	1
	
	Fair	55.7	**2.18 (1.24- 3.82)**	38.3	**1.93 (1.08- 3.43)**	35.0	**2.38 (1.44- 3.94)**

**Feeling of loneliness**	No	36.0	1	22.2	1	15.7	1

	Yes	56.6	**2.45 (1.71- 3.51)**	42.6	**2.53 (1.44- 4.43)**	32.1	**2.79 (1.52- 5.12)**

* The models for each socioeconomic and psychosocial variable are adjusted for age and gender

The analysis of the associations between the migration variables and the health variables (Table 3) shows that, after adjustment for age and gender, the temporary documented immigrants and undocumented immigrants reported poor perceived health more often than the native-born French (Model 1: OR = 2.32; 95% CI = [1.05-5.11] and OR = 3.08; 95% CI = [1.21-7.84]). On the other hand, people born French outside French Guiana reported better perceived health (Model 1: OR = 0.36; 95% CI = [0.16-0.79]). These associations were no longer statistically significant after adjustment for the socioeconomic conditions, but the strengths of association remained rather stable. The naturalized immigrants and undocumented immigrants reported a chronic disease more often, even after their socioeconomic status was taken into account (Model 2: OR = 2.00; 95% CI = [1.06-3.78] and OR = 2.39; 95% CI = [1.05-5.45], respectively). The addition of the psychosocial variables to the model did not cause the strengths of association to change substantially, even if the latter were no longer significant. The undocumented immigrants reported more functional limitations than the native-born French (Model 1: OR = 2.17; 95% CI = [1.00-4.70]), and the associations decreased sharply after the covariables were added (Model 3: OR = 1.00; 95% CI = [0.33-3.07]). The immigrants born in Surinam reported functional limitations more often (Model 1: OR = 2.19; 95% CI = [1.21-3.95]). This association did not persist after adjustment in Models 2 and 3. However, after adjustment for the socioeconomic conditions and the psychosocial variables, the immigrants born in Haiti reported functional limitations significantly less often (Model 3: OR = 0.44; 95% CI = [0.25-0.76]). The immigrants who had been in French Guiana for more than 5 years reported poorer perceived health (Model 1: OR = 2.08; 95% CI = [1.01-4.25]). The strength of this association decreased with the successive adjustments and was no longer significant in Models 2 and 3. On the other hand, after adjustment for the socioeconomic conditions and the psychosocial variables, the immigrants who had lived in French Guiana for 5 years or less reported a functional limitation less often (Model 3: OR = 0.14; 95% CI = [0.02-0.98]).

**Table 3:** Logistic regression models explaining the health variables according to the migration variables (OR and 95% CI)

		Poor self-assessed health			Chronic disease			Functional limitation		
		**Model**	**Model**	**Model**	**Model**	**Model**	**Model**	**Model**	**Model**	**Model**
		**1**	**2**	**3**	**1**	**2**	**3**	**1**	**2**	**3**

**Migration status**	Native-born French	1	1	1	1	1	1	1	1	1

	Born French outside French Guiana	**0.36 (0.16- 0.79)**	0.58 (0.25-1.34)	0.53(0.22-1.27)	0.56(0.19-1.64)	0.66(0.25-1.71)	0.59(0.22-1.58)	0.41(0.12-1.35)	0.50(0.11-2.24)	0.46(0.09-2.27)

	Naturalized immigrant	1.33(0.62-2.87)	1.26(0.59-2.72)	1.21(0.58-2.55)	**1.89(1.09**-**3.28)**	**2.00(1.06**-**3.78)**	1.87(0.89-3.93)	1.41(0.63-3.16)	1.34(0.54-3.35)	1.13(0.46-2.78)

	Long-term documented immigrant	1.43(0.68-3.01)	1.06(0.53-2.11)	1.09(0.53-2.27)	1.45(0.61-3.43)	1.36(0.58-3.17)	1.37(0.57-3.28)	1.13(0.72-1.76)	0.99(0.62-1.58)	0.91(0.54-1.54)

	Temporary documented immigrant	**2.32(1.05**-**5.11)**	1.69(0.81-3.55)	1.74(0.81-3.73)	1.39(0.71-2.75)	1.21(0.60-2.47)	1.14(0.53-2.47)	0.97(0.30-3.07)	0.71(0.18-2.82)	0.50(0.13-1.97)

	Undocumented immigrant	**3.08(1.21**-**7.84)**	2.06(0.89-4.74)	2.16(0.83-5.60)	**2.79(1.22**-**6.34)**	**2.39(1.05**-**5.45)**	2.24(0.82-6.11)	**2.17(1.00**-**4.70)**	1.45(0.57-3.72)	1.00(0.33-3.07)

**Country of birth**	Native-born French	1	1	1	1	1	1	1	1	1

	Born French outside French Guiana	**0.37(0.17**-**0.78)**	0.61(0.27-1.41)	0.56 (0.23-1.34)	0.56(0.20-1.61)	0.66(0.25-1.71)	0.59(0.22-1.57)	0.41(0.12-1.36)	0.49(0.10-2.39)	0.45(0.09-2.36)

	Immigrant born in Haiti	2.22(0.92-5.38)	1.31(0.57-2.99)	1.29 (0.53-3.13)	1.89(0.68-5.23)	1.56(0.59-4.12)	1.51(0.54-4.25)	0.83(0.57-1.21)	**0.50(0.32**-**0.77)**	**0.44(0.25**-**0.76)**

	Immigrant born in Surinam	2.07(0.93-4.60)	1.40(0.70-2.83)	1.50(0.68-3.28)	2.06(0.85-4.99)	1.87(0.69-5.07)	1.88(0.57-6.21)	**2.19(1.21**-**3.95)**	1.83(0.79-4.21)	1.42(0.66-3.05)

	Immigrant born elsewhere	1.74(0.82-3.69)	1.60(0.81-3.18)	1.58(0.81-3.11)	1.56(0.98-2.46)	1.60(1.00-2.58)	1.50(0.86-2.61)	1.54(0.61-3.84)	1.65(0.61-4.48)	1.39(0.55-3.50)

**Durationof residence**	Native-born French	1	1	1	1	1	1	1	1	1

	Born French outside French Guiana	**0.37 (0.17- 0.79)**	0.61(0.27- 1.39)	0.56(0.24- 1.32)	0.57(0.20- 1.60)	0.67(0.26- 1.69)	0.59(0.23- 1.54)	0.41(0.13- 1.31)	0.51(0.12- 2.14)	0.46(0.10- 2.11)

	Established immigrant (> 5 years)	**2.08(1.01**-**4.25)**	1.56(0.78-3.13)	1.54(0.75-3.16)	1.92(1.00-3.70)	1.84(0.97-3.47)	1.74(0.85-3.54)	1.47(0.84-2.57)	1.32(0.71-2.48)	1.07(0.58-1.95)

	Recentimmigrant (≤ 5 years)	1.55(0.73-3.30)	1.04(0.52-2.05)	1.02(0.47-2.26)	1.09(0.34-3.45)	0.79(0.28-2.23)	0.70(0.22-2.17)	0.47(0.10-2.18)	0.20(0.04-1.04)	**0.14(0.02**-**0.98)**

Model 1: Adjusted for age and gender.

Model 2: Adjusted for age, gender, city and socioeconomic variables (education level, occupational status and perceived financial situation).

Model 3: Adjusted for age, gender, city, socioeconomic variables and psychosocial variables (fluency in French and social isolation).

Table 4 shows that the associations between health and being an undocumented immigrant were sometimes very different, depending on the individual's country of birth. Undocumented immigrants born in Surinam reported more functional limitations than the native-born French (Model 3: OR = 3.04; 95% CI = [1.02-9.03]). On the other hand, undocumented immigrants born in Haiti reported fewer functional limitations than the native-born French, regardless of which adjustments were made (Model 3: OR = 0.13; 95% CI = [0.05-0.36]). In addition, this table suggests that the undocumented immigrants born in Surinam had poor health indicators, regardless of which health indicator was used or which adjustments were made: all models combined, the ORs associated with poorer health varied, for this subgroup, from 2.40 (95% CI = [0.91-6.34]) to 4.29 (95% CI = [1.55-11.84]).

**Table 4:** Logistic regression models explaining the health variables according to migration status and country of birth (OR and 95% CI)

		Poor self-assessed health			Chronic disease			Functional limitation		
		Model	Model	Model	Model	Model	Model	Model	Model	Model
		1	2	3	1	2	3	1	2	3

**Migration status and country of birth**	Native-born French	1	1	1	1	1	1	1	1	1
	
	Born French outside French Guiana	**0.36(0.16**-**0.79)**	0.58(0.25-1.34)	0.53(0.22-1.28)	0.56(0.19-1.64)	0.66(0.26-1.71)	0.59(0.22-1.57)	0.41(0.12-1.35)	0.49(0.11-2.23)	0.44(0.09-2.27)
	
	Naturalized immigrant	1.33 (0.61- 2 .88)	1.26(0.58-2.76	1.22(0.57-2.60)	1.89(1.09-3.29)	2.00(1.05-3.80)	1.89(0.90-3.96)	1.40(0.62-3.16)	1.29(0.51-3.24)	1.11(0.44-2.82)
	
	Long-term documented immigrant	1.43(0.68-3.01)	1.07(0.55-2.10)	1.12(0.55-2.25)	1.45(0.61-3.43)	1.39(0.59-3.29)	1.44(0.60-3.45)	1.12(0.72-1.75)	1.02(0.64-1.63)	0.98(0.58-1.65
	
	Temporary documented immigrant	**2.32(1.05**-**5.11)**	1.70(0.82-3.53)	1.78(0.87-3.66)	1.39(0.70-2.75)	1.23(0.61-2.51)	1.21(0.56-2.61)	0.97(0.30-3.08	0.73(0.17-3.03)	0.56(0.14-2.31)
	
	Undocumented immigrant born in Surinam	**3.29(1.11**-**9.70)**	2.40(0.91-6.34)	2.82(0.99-8.40)	**3.79(1.73**-**8.31)**	**4.07(1.80**-**9.22)**	**4.29(1.55**-**11.84)**	**4.20(1.89**-**9.33)**	**4.17(1.30**-**13.39)**	**3.04(1.02**-**9.03)**
	
	Undocumented immigrant born in Haiti	3.00(0.63-14.22)	1.63(0.33-8.01	1.54(0.31-7.74)	1.60(0.35-7.20)	1.22(0.27-5.57)	1.12(0.23-5.51)	**0.37(0.16**-**0.83)**	**0.16(0.06**-**0.42)**	**0.13(0.05**-**0.36)**
	
	Undocumented immigrant born elsewhere	2.72(0.57-12.88)	2.35(0.55-9.98)	2.79(0.59-13.13)	2.94(0.73-11.80)	2.52(0.61-10.35)	2.57(0.53-12.44)	1.97(0.30-13.02)	1.89(0.36-9.89)	1.34(0.16-10.95)

Model 1: Adjusted for age and gender.

Model 2: Adjusted for age, gender, city and socioeconomic variables (education level, occupational status and perceived financial situation).

Model 3: Adjusted for age, gender, city, socioeconomic variables and psychosocial variables (fluency in French and social isolation).

## Discussion

To the best of our knowledge, this study is the first one carried out in French Guiana that describes and analyzes social and health disparities in specific populations on the basis of their origins and migration status. This study shows that these two cities have large immigrant populations (40.5% of the adult population in Cayenne and 57.8% in Saint-Laurent du Maroni) and that many of these individuals' had been there for long while (60.7% of the immigrants living in Cayenne and 77.5% in Saint-Laurent du Maroni had been living there for more than 10 years). Despite this long duration of residence in French Guiana, a substantial portion of the immigrant population had no stay documents or had a precarious status. An analysis of the population's social and economic conditions shows strong inequalities that follow a gradient according to the individual's legal status with regard to his or her stay. The analyses showed that the health of these populations depends on several migration-related factors, but also on how health is measured. Three key findings are noted. First, in general, of all the subgroups of migrants that were studied, those most vulnerable and with the worst health status were those who were undocumented, regardless of which social and health indicators were considered. Second, when health was measured as perceived health, the analyses showed that the undocumented immigrants and the documented immigrants with a precarious status (with a stay document valid for one year or less) reported poorer perceived health than the native-born. The country of origin and the duration of residence did not change these results very much. These observed associations are only partially explained by the individuals' socioeconomic status. Third, with regard to functional limitations, certain groups of immigrants (recent immigrants and those born in Haiti) reported a more favourable situation than the native-born for a comparable socioeconomic status.

Although the literature on this topic is sparse, several studies and reports suggest it is undocumented immigrants who are the most vulnerable with regard to health [1,13-18]. They suffer from a combination of socioeconomic conditions and working conditions that are precarious or even harmful to their health [19], and they have difficulty accessing health care. In our study, it was mainly the undocumented immigrants who seemed to be the worst off socioeconomically. The socioeconomic indicators used in this study explain only some the observed differences in health. The remaining differences could be explained by socioeconomic factors that were not taken into account in this study (such as income, working conditions or housing conditions) and by difficulty accessing health care. In French Guiana, as in mainland France, undocumented immigrants can theoretically access health care free of charge through a specific health insurance system called "*Aide Médicale État*" (government medical assistance, which is government-run, unlike the usual health insurance system, which is run by Social Security). If, as several reports have shown, there is, in France, a gap between theoretical rights and actual rights to health care (due to the complexity of the system, the difficulty people have in presenting the required administrative documents, the lack of information on the part of administrative personnel, differences in their practices, and so on [15,16]), then these difficulties are surely much worse in French Guiana [20,21].

Our results for perceived health are consistent with those of several international and French studies. A systematic review with the objective of examining and comparing self-perceived health among migrants and ethnic minority groups in EU countries showed that most migrants and ethnic minority groups appeared to be disadvantaged in relation to the majority population, even after controlling for age, gender and socioeconomic factors [22]. A study carried out in mainland France among a sample of more than 20,000 people that was representative of the general population (Enquête décennale santé [Decennial Health Survey]) found that people of foreign origin living in France reported poorer health than the French born in France. It did not find any differences in health between foreign immigrants and those who had been naturalized. As in our study, these populations' poor socioeconomic conditions only partially explained their poorer perceived health [23]. A study carried out on Mayotte Island, a French overseas territory in the Comoros Archipelago, found that the health of foreigners was less good there as well (and they were found to have more difficulty accessing health care) than that of the French [24].

The recent immigrants to French Guiana (≤ 5 years) reported fewer functional limitations than the native-born French. This finding supports the "healthy immigrant effect" hypothesis, according to which migrants represent a selectively healthy group that is not representative of all potential migrants from origin societies [25-27]. This hypothesis is also supported by additional analyses in this study suggesting that the migration of sick people (or health care migration) accounts for only a minority of migration movements [28]. This is not observed for perceived health, which may be due to the cut-off that was chosen. Indeed, other studies suggest that the decline in self-perceived health occurs over a very short period after migration [29-31]. In addition, several studies have found relatively better health outcomes for immigrants for indicators such as mortality, chronic conditions and impaired activity than for self-assessed health [26,29,32,33], which suggests that health selection is stronger for chronic and severe conditions.

After adjustment for the socioeconomic conditions, the people born in Haiti reported fewer functional limitations than the native-born French. This subgroup of immigrants had the worst socioeconomic indicators (47.2% of the people born in Haiti had no or only a primary education, 60.7% reported that they did not have enough to live on, and only 34.5% were working). Moreover the proportion of recent immigrants (≤5 years) among immigrants born in Haiti (14.2%) was not different from the one among immigrants from other countries (15.5%). Therefore, three hypotheses could explain this paradoxical finding. One is that of cultural differences in reporting functional limitations, although it hardly seems plausible (this hypothesis will be detailed below). Another is that of selection bias due, in this case, to the return of migrants in poor health to their country of origin, which seems even more unlikely, given the overall situation in Haiti. A third hypothesis seems the most probable: that of greater migration selection among migrants from Haiti, the poorest country in the Americas [34]. A recent study carried out in Spain found that "[f]oreign immigrants from poor countries reported the worst socio-economic conditions, but relatively good health" [33]. Other studies suggest that long distance migration may be associated with a stronger selection effect [7,26,35]. It may be that Haitians in better physical health are the ones more likely to move to French Guiana because they are able to manage the difficulties and stress associated with immigrating. The undocumented immigrants from Surinam had poor health indicators, regardless of which health indicator was used or which adjustments were made. These people have special attributes: all of them were living in Saint-Laurent du Maroni (a town on the border with Surinam), and their median duration of residence in French Guiana was 16 years (as opposed to 6 years for the other undocumented immigrants). Thus, a number of hypotheses can be proposed to explain their particularly poor health status: the circumstances of their immigration to French Guiana (fleeing from the civil war between 1986 and 1992 and economic hardships in Surinam), the geographical proximity of Saint-Laurent du Maroni (which limits the possibility of positive immigration-selection bias), and the many years spent underground.

### Limitations and strengths of this study

This study has a certain number of strengths: a sampling method ensuring that the final sample would be representative, a high participation rate, and the inclusion of several migration variables.

Several limitations should be discussed. First, this was a cross-sectional study, and no definite conclusions can be drawn regarding causality. Second, this survey was conducted among people over the age of 18 years who had been living or were intending to live in French Guiana for at least 6 months and who were residing in single-family dwellings. It therefore excluded people living collectively, people with no fixed address, and transient migrants. Third, we did not have a means of measuring the representativeness of the subgroup consisting of undocumented immigrants, since they are, by definition, undocumented in the national statistics. On the other hand, the sampling procedure (the stratification and sampling intervals used) and the large proportion of this population in the two survey cities make it unlikely that we under- or overrepresented the neighborhoods inhabited by undocumented immigrants. Lastly, a few words need to be said about the choice of indicators. The three health indicators of the MEHM had the advantage of being widely used in epidemiological surveys, and their reliability had been evaluated in a European population [12]. However, they have not been validated in the populations of French overseas departments (especially in French Guiana). Moreover, questions remained about their interindividual comparability, since health perceptions vary according to health norms and people's aspirations, who are influenced by their social and cultural environment [36,37]. Of the three health indicators used, a self-reported chronic disease is the most prone to differential reporting bias between social groups [38]. In this study, homemakers and individuals with little schooling reported poor perceived health and functional limitations more often, but these associations were not found for the indicator 'chronic disease'. Several analyses have reported a trend toward chronic diseases in population groups in the lowest education and income brackets being underreported [38-40]. This can be explained by less medical information, which is due to less use of the health-care system. In addition, it is questionable whether the concept of chronic disease is clearly understood by all sociocultural groups. Perceived health is the mostly widely used indicator, and numerous studies have shown associations with mortality [36,41], morbidity and the use of the health-care system [42,43], regardless of the ethnic group [44,45]. However, a few studies found that this indicator tended, once again, to underestimate social health inequalities [38,46]. As for the indicator 'functional limitations', its transcultural validity has not been investigated, but several studies that have examined this indicator between different ethnic groups suggest that information biases are weak [47-50].

The choice of migration variable has its limitations, too. The main one is that the groupings that were made (to construct the six subgroups based on migration status) mask very different sociocultural situations and migration paths. For instance, the subgroup consisting of people born in French Guiana was actually quite heterogeneous (among the main ethnic groups that make up the population in French Guiana are the Creoles, the Bushinenge and Amerindians). Furthermore, when constructing these six groups, we took into account the individual's status on the day of the survey. This categorization did not take into account how long the person had had that status, for some statuses are not stable. Immigrants with a temporary stay document can have their renewal request turned down and quickly become undocumented. In contrast, some of the interviewees may have very recently regularized their status.

## Conclusion

Overall, the results of this study suggest that, although the determinants of migrant health in French Guiana mainly have to do with the multiple dimensions of the social determinants of health and social health inequalities, other parameters specific to immigration (the country of origin, the duration of residence, the reason for immigrating, and the conditions of residence in French Guiana) play a role of their own. Data on migrant health are scarce in France, and more generally, migrant health problems have been largely ignored in public health policies. Indeed, the notion of a "specific approach" to health issues is creating a debate. Any differential treatment, in particular, according to nationality or ethnic group, is generally perceived as wrong, since it is contrary to the principle of equal treatment for all citizens guaranteed by the Constitution and that is part of a long republican tradition. In reality, such a view shows a lack of understanding, all the while contributing to the denial of the problems specific to immigrants. We recommend that the specific determinants associated with migration be taken into account in different epidemiological surveys and the current local information systems to improve knowledge of the health of specific populations in French Guiana. The health status of migrants is of crucial interest to health policy planners, and it is especially relevant, considering the size of the foreign-born population in that region.

## Competing interests

The authors declare that they have no competing interests.

## Authors' contributions

PC and JL conceived this study and supervised all the aspects of its realisation and dissemination. AJ was involved in the study design, data collection and data analysis. SF and EC were involved in the study design. All the authors approved the final manuscript

## Pre-publication history

The pre-publication history for this paper can be accessed here:

http://www.biomedcentral.com/1471-2458/12/53/prepub
